# A Comprehensive Review of Dietary Approaches in Maintaining Remission of Inflammatory Bowel Diseases in Adults

**DOI:** 10.3390/medicina60071068

**Published:** 2024-06-28

**Authors:** Doina Istratescu, Carmen Monica Preda, Teodora Manuc, Corina Meianu, Tudor Stroie, Mircea Diculescu

**Affiliations:** UMF “Carol Davila” Gastroenterology & Hepatology Department, Fundeni Clinical Institute, 022328 Bucharest, Romania; doina.proca08@gmail.com (D.I.); teodora.manuc@gmail.com (T.M.); corina_meianu@yahoo.com (C.M.); stroie.tudor@gmail.com (T.S.); mmdiculescu@yahoo.com (M.D.)

**Keywords:** diet, inflammatory bowel disease, Crohn’s disease, ulcerative colitis, remission

## Abstract

Inflammatory bowel disease (IBD) poses significant challenges in its management, encompassing a spectrum of conditions from Crohn’s disease to ulcerative colitis. Dietary interventions have emerged as integral components of the multidisciplinary approach to IBD management, with implications ranging from disease prevention to treatment of active manifestations and addressing complications such as malnutrition. While dietary interventions show promise in improving outcomes for some patients with IBD, there is no consensus in the existing literature regarding remission maintenance in those patients. Furthermore, many patients explore dietary modifications often guided by anecdotal evidence or personal experiences and this could lead to malnutrition and decreased quality of life. This comprehensive review synthesizes existing literature to elucidate the complex interplay between diet and IBD, offering insights into the efficacy and safety of various dietary modalities in maintaining disease remission. It also highlights the importance of patient education in navigating dietary choices and potential risks associated with food avoidance, including the heightened risk of micronutrient deficiencies. Furthermore, it emphasizes the pivotal role of a multidisciplinary care team comprising clinicians and dietitians in providing personalized dietary guidance tailored to individual patient needs and goals. By synthesizing the latest evidence and providing insights into both the potential benefits and risks of dietary interventions, this review could be used as a resource for healthcare professionals and patients alike in navigating the complex landscape of dietary management in IBD.

## 1. Introduction

Inflammatory bowel diseases (IBDs) are a heterogeneous group of chronic inflammatory conditions that include Crohn’s disease (CD) and ulcerative colitis (UC). CD is a transmural inflammatory disease that can affect any component of the gastrointestinal tract, while UC is limited to the mucosa and affects only the colon. Both diseases exhibit a relapsing-remitting clinical course characterized by periods of active disease and clinical remission [1,2,3].

The elucidation of the etiology of IBD continues to be an area of active investigation. The prevailing conceptual model suggests a nuanced interplay involving an aberrant immune response, environmental influences, and the intricate dynamics of the gastrointestinal microbiota, particularly in individuals with genetic susceptibilities to the disorder [4,5,6]. The escalating incidence of IBD in recently industrialized countries adopting Western lifestyles underscores the significant influence of environmental factors, particularly dietary patterns, on the onset and progression of IBD [7,8]. The global prevalence in 2019 was estimated to be 4.90 million IBD cases, with the largest number being observed in Western Europe [9].

While epidemiological evidence indicates that specific dietary components may play a role in the onset of IBD, the precise foods that influence disease progression and exacerbations remain uncertain. Various elements within diets have been suggested as potential exacerbating factors with anti-nutritive properties, encompassing processed foods and their associated additives [10,11]. Thus far, several dietary regimens, have been investigated in patients with IBD. However, these studies exhibit considerable heterogeneity in their design and the level of supporting evidence available [12].

Despite significant progress in understanding the pathogenesis of IBD and the development of novel therapeutic approaches, its clinical management remains complex. The current standard of care for IBD primarily relies on immunosuppressive medications and biological therapies, yet their effectiveness is variable and often accompanied by significant adverse effects and risks. As a result, there is increasing interest in investigating the potential role of dietary interventions within the therapeutic landscape for managing IBD [13].

Exclusive enteral nutrition (EEN), recommended by the European Crohn’s and Colitis Organisation (ECCO) and the European Society for Paediatric Gastroenterology Hepatology and Nutrition (ESPGHAN), serves as a prominent dietary intervention for inducing remission in newly diagnosed, mild to moderate CD among pediatric patients [14]. Attempts have been made to extend the application of EEN to adult CD populations. Comparative research between EEN and corticosteroids yielded comparable outcomes in adults completing the treatment compared to the pediatric cohort. However, non-adherence to EEN, affecting up to 50% of participants, is a concern due to factors like low motivation, insufficient support, and the unpalatability of the formula [15,16]. Consequently, dietary regimens integrating conventional food products may offer improved tolerability for patients.

Furthermore, aside from comprehending the mechanisms facilitating remission initiation, it is crucial to identify methodologies for maintaining remission via dietary intervention. A recent study unveiled a rapid surge in fecal calprotectin (FC) concentrations within the initial 2–3 weeks subsequent to reintroducing food in children who had attained remission through EEN [17]. Consequently, the reintroduction of dietary constituents upon transitioning to a standard diet is associated with elevated FC levels and the reactivation of intestinal inflammation [4].

Patients diagnosed with IBD often express significant concerns regarding their dietary choices and nutritional intake. In an effort to alleviate gastrointestinal discomfort and improve their overall well-being, they frequently adopt a variety of dietary strategies [18,19]. Research indicates that up to 71% of individuals with IBD perceive their diet as a key factor influencing their disease symptoms. This perception leads a considerable number of patients, including 90% of those with CD and 71% of those with UC, to implement elimination diets during periods of remission [20,21]. However, altering dietary habits may give rise to apprehensions, particularly if patients significantly restrict or completely eliminate essential nutrients or food groups, potentially increasing their susceptibility to nutritional deficiencies and reducing their overall quality of life. Notably, a significant proportion, up to 77.1%, of individuals with IBD report avoiding specific food items [20,22]. Considering the persistent efforts of patients to modify their dietary patterns in an effort to ameliorate their condition, the identification of potential causative dietary factors is a central focus in IBD research at the moment.

This narrative review aims to explore the evidence surrounding the effectiveness of commonly explored dietary strategies and specific dietary patterns in upholding remission in adult patients with IBD. We outline the inherent strengths and limitations of the prevailing body of evidence, providing healthcare practitioners with a systematic framework for guiding patients on dietary interventions. This framework serves as a foundation for future research aimed at investigating the role of diet in extending clinical remission in IBD.

## 2. Material and Methods

A literature review was conducted on databases, including PubMed, with “advanced” and “MeSH” tools, using the following key term queries: (inflammatory bowel disease AND diet AND remission) [title]; (crohn’s disease AND diet AND remission) [title]; (ulcerative colitis AND diet AND remission) [title]; inclusion criteria were as follows: narrative and systematic reviews, clinical trials, meta-analyses, and randomized controlled trials, published in the last 10 years, giving priority to the most recent. Exclusion criteria were as follows: clinical case studies, letters or editorials of magazines, abstracts to conferences, book chapters, and unpublished materials.

## 3. The Importance of Diet in IBD

The correlation between diet and IBD may commence as early as infancy. Findings from a meta-analysis conducted in 2017 provided evidence supporting the protective role of breastfeeding in reducing the risk of IBD development, with the strongest protective effect observed among individuals breastfed for at least 12 months [23]. The early phases of growth and the initiation of dietary intake represent a critical period for the intestinal microbiome, with potential implications for the integrity of the intestinal mucosa and immune responses to luminal contents. Additionally, the presence of immunoglobulins in breast milk may provide defense against enteric infections, thereby fostering a balanced microbiome. The introduction of solid foods represents another noteworthy event in gut microbiome health and bacterial diversity, highlighting the ongoing influence of diet on growth and development [24,25,26].

Currently, there are distinct dietary patterns believed to worsen symptoms in individuals diagnosed with IBD, as well as others associated with improving symptoms over both short and long durations [27].

The Western diet has been demonstrated to alter the intestinal microbiota in healthy people, being marked by increased consumption of refined sugars, omega-6 polyunsaturated fats, and fast food, coupled with inadequate intake of fruits, vegetables, and fiber. A considerable portion of modern food sources undergo substantial processing, alteration, and transportation over long distances, diverging from traditional dietary practices where locally sourced food is consumed soon after harvesting [21,28].

This shift towards the Western dietary pattern frequently results in reduced microbial diversity and discernible shifts in microbial composition [29,30]. Furthermore, it facilitates the proliferation of mucin-degrading bacteria such as *Bacteroides thetaiotaomicron* and *Akkermansia muciniphila* [31]. Additionally, the consumption of a high-fat and high-sugar diet has been associated with an increased abundance of *Proteobacteria* and adherent-invasive Escherichia coli, while *Firmicutes* were more prevalent in mice fed a conventional diet [29]. Mice subjected to a high-fat and high-sugar diet also displayed heightened levels of *Bacteroides* spp. and *Ruminococcus torques* [32]. Consequently, there was a decline in the production of short-chain fatty acids (SCFAs) by the intestinal microbiota, coupled with a decrease in the population of regulatory T cells (Tregs) in the mesenteric lymph nodes [30]. These changes have been correlated with compromised intestinal mucus integrity and a heightened susceptibility to IBD.

According to more recent prospective cohort studies, individuals with IBD are advised to avoid consumption of sugar-sweetened beverages, given their association with an increased risk and the worsening of a more severe long-term clinical course of the disease [33,34].

The Mediterranean diet is suggested to enhance the diversity of the gut microbiome and metabolome through mechanistic pathways, potentially providing long-term health benefits, such as reducing the risk of cardiovascular disease, metabolic syndrome, and cancer. Chicco et al. [35] conducted an independent study to validate the efficacy of the Mediterranean diet specifically tailored for patients with IBD. Nutritional guidance was provided to 142 individuals diagnosed with IBD. After a six-month intervention period, both UC and CD patients adhering to the Mediterranean diet showed decreased disease activity rates, lower levels of inflammatory biomarkers, and improved overall quality of life. Adhering to a wholesome and comprehensive Mediterranean diet provides the added benefit of effectively reducing the intake of heavily processed foods, which commonly contain extra sugar, excessive salt, and various food additives [27].

## 4. Nutrition Assessment in IBD Patients

The importance of assessing the nutritional status of IBD patients resides in the association of common deficits with an unfavorable course of the disease. Malnutrition and a spectrum of nutritional deficiencies are common but frequently overlooked complications in IBD, notably prevalent among individuals with CD and those who have undergone repeated surgical procedures. Malnutrition is associated with unfavorable consequences in IBD, such as heightened occurrences of emergency department visits, prolonged hospital stays, non-elective surgeries, increased mortality rates, reduced responsiveness to medical treatments, and compromised overall quality of life [36,37,38].

The European Society for Parenteral and Enteral Nutrition (ESPEN), the Academy of Nutrition and Dietetics, and the American Society for Parenteral and Enteral Nutrition advocate for the systematic screening of malnutrition in individuals diagnosed with IBD both at the time of diagnosis and at regular intervals during ongoing management [26]. Patients with IBD should undergo regular malnutrition assessments using validated tools such as the Nutritional Risk Screening 2002 [39] or the Malnutrition Universal Screening Tool [40]. Furthermore, diagnostic assessment based on the Global Leadership Initiative on Malnutrition (GLIM) criteria [41] should be performed when warranted, aligning with guidelines set forth by the global clinical nutrition community. This assessment requires meeting one positive etiological criterion, usually fulfilled by the presence of the underlying inflammatory condition, in addition to one phenotypical criterion. The latter encompasses manifestations such as “non-volitional weight loss”, “low body mass index (BMI)”, or “reduced muscle mass” [40]. It is essential to recognize that although a low BMI can be indicative, the presence of a malnourished phenotype may be evident regardless of body morphology, encompassing individuals with lean, normal, or obese body compositions.

Malnutrition in patients diagnosed with IBD largely arises from various factors, including diminished oral intake, increased requirements for energy and protein, and amplified gastrointestinal losses resulting from inflammatory processes, malabsorption, disease activity, short bowel syndrome (SBS), and the use of specific medications. Additionally, a significant portion of individuals with IBD exhibit a heightened tendency towards food avoidance (28–89%) and adopt restrictive dietary behaviors (41–93%), profoundly affecting their food-related quality of life (QoL). The main factors that contribute to the development of malnutrition in IBD are presented in Table 1 [42,43].

This inclination exposes patients to an elevated risk of developing nutritional deficiencies, both during active disease phases and periods of remission, as previously evidenced by nutrients such as vitamin C, copper, niacin, zinc, and others [44,45]. In a nationally representative sample of hospitalized individuals, the probability of malnutrition was 5.57 times higher in patients diagnosed with IBD compared to those without the condition [38]. Among patients receiving care in outpatient clinics specializing in IBD management, the overall prevalence of malnutrition was 16%, with a significant majority of malnourished individuals being diagnosed with Crohn’s disease (56.8%) [37,46].

Deficiencies in micronutrients like vitamin D, iron, and vitamin B12 are commonly encountered in individuals diagnosed with IBD. These deficiencies may stem from various factors, including chronic inflammation of the mucosal lining, stringent dietary restrictions, prolonged periods of bowel rest, malabsorption issues, changes in bowel structure affecting length and absorptive capacity, and interactions between medications and nutrients [47,48]. Furthermore, depending on specific risk profiles, it is important to assess and address potential deficiencies in other vital vitamins and minerals such as zinc, copper, fat-soluble vitamins, and folic acid, especially in patients undergoing treatment with methotrexate and sulfasalazine [47,48].

To conclude, upon recognizing malnutrition and confirming a diagnosis, the extent of malnutrition can be assessed and augmented through a comprehensive evaluation to devise a customized care plan that caters to individual nutritional needs. For example, if a deficiency in a specific nutrient like iron, vitamin C, or folic acid is detected, supplementation can be administered via medication or adjustments to the diet (Table 2) [47,48].

Therefore, the principal aim of a personalized dietary regimen should be dual-fold to assess nutritional status and avoid nutritional deficiencies.

## 5. Dietary Approaches in Maintaining IBD Remission

Multiple dietary interventions were evaluated in maintaining remission in IBD adult patients, their characteristics being described in Table 3 and further detailed in Section 5.1 and Section 5.2.

### 5.1. Specific Dietary Interventions

#### 5.1.1. The Crohn’s Disease Exclusion Diet (CDED)

The Crohn’s Disease Exclusion Diet (CDED) is structured as a three-phase, step-down dietary regimen. The initial phase spans 6 weeks and incorporates partial enteral nutrition (PEN) supplying 50% of nutritional requirements, alongside the inclusion of 14 specific foods containing protein, resistant starch, or pectin. The underlying hypothesis of the CDED posits that dietary elements emblematic of a Western diet (characterized by low fiber content and high levels of fat, sugar, and additives) exert detrimental effects on the gut microbiota, thus contributing to the onset of Crohn’s disease [63].

The therapeutic efficacy of the PEN component remains uncertain. An open-label pilot trial involving 44 adults diagnosed with mild ileal Crohn’s disease revealed comparable clinical responses among those following the CDED, irrespective of whether they received PEN. This conclusion was drawn based on assessments of C-reactive protein (CRP) levels, fecal calprotectin levels, and clinical disease scores [49]. The proposed third phase of CDED, which involves a broader range of food choices without the use of formula, has not yet been assessed for safety, particularly regarding its impact on nutrition in the absence of partial enteral nutrition (PEN) support. There are concerns regarding potential adverse effects on both nutrition and psychosocial well-being if phase 3 is recommended for ongoing use [16,64].

#### 5.1.2. Plant-Based Diet

Plant-based diet involves the limitation of animal fats and proteins, representing a semi-vegetarian dietary approach. It also entails restrictions on sweets, bread, cheese, margarine, fast foods, carbonated beverages, juices, and alcohol, with fish intake limited to half portions per week and meat consumption restricted to half portions every two weeks. The rationale behind adopting a plant-based diet is that a high intake of prebiotics can promote the growth of beneficial colonic bacteria, thereby reducing inflammation in IBD. Chiba et al. [51] demonstrated a reduced risk of relapse over a two-year period in 16 patients with Crohn’s disease (CD) who were in clinical remission. There are concerns regarding nutritional adequacy that have not been assessed.

#### 5.1.3. Low-Fat Diet

*Low-fat diet* limits fat intake to 10% of total calories and reduces red meat consumption to half a serving per day, while also incorporating a total of 25 grams of dietary fiber. This dietary approach is founded on the observed elevated risk of developing ulcerative colitis (UC) associated with diets high in fat and animal meat. Fritsch et al. [52], in a cross-over study involving 17 patients with UC in remission, identified a positive correlation between adherence to a low-fat diet and reductions in markers of inflammation and intestinal dysbiosis. A significant advantage of this dietary regimen is its safety profile, as it does not lead to nutritional deficiencies or adversely affect psychosocial well-being.

#### 5.1.4. Low-Meat Diet

The *low-meat diet*, referred to as FACES (Frequency of Animal Consumption, Exclusion of Certain Foods, and Symptom Monitoring), restricts the consumption of red and processed meats to one serving per month or less. This dietary regimen operates under the premise that reducing meat intake may help prevent disease relapse in individuals with quiescent Crohn’s disease. However, findings from the FACES trial conducted by Albenberg et al. [53] revealed no discernible association between the level of red and processed meat consumption and the time to symptomatic relapse in patients with Crohn’s disease in remission.

#### 5.1.5. IgG-Based Food Exclusion

The *IgG-based food exclusion* diet is a six-month intervention that involves restricting foods based on the presence of IgG antibodies specific to 14 food antigens, including egg, wheat, milk, corn, rice, soybean, and chicken, among others. An observational study conducted by Jian et al. [54] involving 97 patients with ulcerative colitis, either in remission or experiencing mild to moderately active disease, compared the effects of an open-label IgG exclusion diet with those of an unchanged diet. The study found that the exclusion diet group exhibited lower stool frequency compared to the unchanged diet group, with no discernible differences observed during endoscopic examination. Additionally, there was no notable increase in IgG antibodies in response to food in symptomatic individuals compared to healthy asymptomatic individuals. However, concerns regarding nutrition arise due to the potential restriction of multiple allergens.

#### 5.1.6. Anti-Inflammatory Diet

An *anti-inflammatory diet* characterized by foods rich in dietary fiber, prebiotics (fructans and galactooligosaccharides), antioxidants (fruits and vegetables), probiotics (fermented foods), and omega-3 polyunsaturated fatty acids (found in oily fish), while limiting intake of red meat, sugar, and alcohol, was evaluated in a randomized controlled trial involving 28 patients with ulcerative colitis (UC) in remission, albeit with a high risk of bias [55]. The study revealed comparable relapse rates and levels of fecal calprotectin at 6 months between participants following the anti-inflammatory diet (5 out of 14) and those receiving dietary advice based on Canada’s Food Guide (4 out of 14). Notably, the group adhering to Canada’s Food Guide experienced a significant increase in fecal calprotectin levels over the six-month period, whereas the anti-inflammatory diet group did not exhibit such an increase.

#### 5.1.7. Dietary Exclusion Regimen

A *dietary exclusion regimen*, which entails reducing intake of disaccharides, saturated fats, emulsifiers, red meats, and ultra-processed meats, was examined for its efficacy in maintaining remission in patients with both UC and CD over a period of six months. Nitescu et al. [56] found that among 139 individuals with IBD who adhered to the exclusion diet for the specified period, there was a higher rate of remission. However, there are concerns regarding the nutritional status of these patients, as it was not assessed in the study.

### 5.2. Enteral Nutrition

Enteral nutrition (EN) is recognized as a well-established and minimally invasive therapeutic strategy with a low-risk profile, exhibiting effectiveness in the treatment of IBD. Exclusive enteral nutrition (EEN) involves the consumption exclusively of liquid formulas, omitting solid foods, typically administered over a period of 6–8 weeks, and capable of satisfying all daily nutritional requirements. Additionally, enteral nutrition can be administered in the form of partial enteral nutrition (PEN), entailing the replacement of 35–50% of regular food intake with EN [57].

To sustain remission in patients with CD, several randomized controlled trials (RCTs) assessed the effectiveness of PEN [58,59,60,61,62]. Pooling data from these studies revealed that a greater proportion of patients who received PEN were able to maintain remission compared to those who did not. This conclusion was drawn from the evaluation of relapse rates predominantly among adult CD patients who were in remission at the outset of the studies.

When enteral nutrition (EN) is utilized alongside biologic therapy, there is evidence suggesting improved outcomes. A meta-analysis of studies involving adults diagnosed with Crohn’s disease (CD) demonstrated the benefits of partial enteral nutrition (PEN) when administered concurrently with maintenance infliximab. This combined treatment approach led to extended periods of clinical remission, with sustained one-year remission rates reaching 75% in the infliximab/PEN group, contrasting with a rate of 49% observed in the infliximab monotherapy group [65].

There is insufficient evidence to prove the efficacy of EEN as a therapeutic approach for both pediatric and adult UC [66].

### 5.3. Probiotics and Prebiotics

Three studies have investigated the efficacy of probiotics compared to placebo in maintaining disease remission for periods ranging from 4 weeks to 12 months in CD [67,68,69]. Across these studies, none demonstrated a reduction in relapse rates with probiotic use (39%) compared to placebo (40%). Similarly, four studies have evaluated probiotics versus placebo for maintaining disease remission in UC. Of these, one study reported a lower relapse rate with probiotics (20%) compared to placebo (93%) after 8 weeks [70], while the remaining studies found comparable relapse rates between probiotics at 4 weeks [69] and 12 months [71,72].

The role of prebiotics in UC remission was assessed by Sinopoulou et al. [73] in a meta-analysis and they found no difference in the occurrence of clinical relapse when adjuvant treatment with prebiotics is compared with adjuvant treatment with placebo.

### 5.4. Complementary and Alternative Medicine

In one RCT, the efficacy of 2 grams per day of curcumin versus placebo was investigated over a period of 6 months in patients with quiescent UC. The study revealed a significantly lower relapse rate among patients receiving curcumin supplementation (5%) compared to those receiving placebo (21%) (*p* = 0.04). Notably, curcumin was administered in conjunction with standard medication (sulfasalazine or mesalamine) [74]. In another RCT involving 62 post-operative patients with CD who were receiving azathioprine, curcumin was found to be no more effective than placebo in preventing endoscopic or clinical recurrence of Crohn’s disease at the six-month mark [75].

### 5.5. Vitamin D

As a standard health practice, vitamin D levels should be monitored and supplemented in individuals with deficiencies. In one study, 6 out of 46 patients (13%) who were taking 1200 IU per day of vitamin D experienced a relapse, compared to 14 out of 48 patients (29%) taking a placebo (*p* = 0.056) [76]. In another study, 6 out of 18 patients (33%) taking 10,000 IU per day experienced a relapse, whereas 11 out of 16 patients (69%) taking 1000 IU per day experienced a relapse [77].

## 6. Conclusions

Diet plays a crucial role in the comprehensive management of IBD across its spectrum, encompassing prevention, treatment of active disease, and addressing complications such as malnutrition. Previous research has shed light on the intricate interplay between diet and IBD. Given patients’ interest in dietary interventions for managing IBD, there is a tendency for individuals to self-impose food restrictions, potentially increasing the risk of micronutrient deficiencies. Comprehensive patient education provided by a multidisciplinary IBD care team comprising clinicians and dietitians, along with the development of personalized dietary plans, can lead to favorable outcomes from both provider and patient perspectives.

Currently, there is no dietary therapy recommended specifically for maintaining remission in IBD. Nonetheless, despite limited empirical support, many patients still experiment with various dietary approaches. While some diets lacking robust evidence for improving outcomes in individuals with irritable bowel syndrome (IBS) may offer benefits for certain patients, there are inherent risks associated with restrictive diets, including potential impacts on nutrition and psychological well-being. Moreover, several diets may inadvertently promote unhealthy or disordered eating behaviors, particularly due to the absence of defined timeframes for their use.

Dietary guidance must be tailored to the unique nutritional status and goals of each IBD patient, which may evolve over time. The implementation of more intricate nutritional strategies for managing IBD is most effectively achieved through collaborative interdisciplinary efforts between gastroenterologists and dietitians.

## Figures and Tables

**Table 1:** Factors responsible for IBD malnutrition.

Factor	Mechanism
**Poor dietary intake**	- **Anorexia triggered by inflammation:** high levels of IL-6 and TNF-α are responsible for cachexia - **Dietary restrictions due to symptoms or medical advice:** some patients adopt a restrictive diet due to symptomatic disease, personal beliefs or even medical advice without a proper nutritional approach - **Gastrointestinal symptoms like nausea, vomiting, and abdominal pain:** some patients limit proper food intake in order to ameliorate their symptoms - **Obstruction of the digestive tract caused by inflammation:** obstructive symptoms (nausea, vomiting) result in dietary restrictions such as solid food intake for several days - **Side effects of medications used for managing IBD symptoms:** nausea, abdominal pain or vomiting impair adequate nutrition in some patients
**Increased nutrient malabsorption**	- **Inflammation affecting nutrient absorption:** mucosal inflammation of the jejunum - **Surgical procedures limiting nutrient absorption:** extensive resection surgeries of the small intestine - **Diarrhea disrupting absorption processes:** impaired absorption of bile acids, changes in microbiota, alterations in lipid digestion and steatorrhea - **Overgrowth of bacteria in the gut interfering with absorption:** changes in microbiota, alterations in lipid digestion and steatorrhea - **Malabsorption of bile acids:** ileal resection, impaired absorption due to inflammation
**Increased protein loss**	- **Inflammation of the intestinal lining (mucosa):** increased levels of inflammatory cytokines disturb intestinal barrier function and loose proteins through capillary leakage - **Formation of abnormal connections (fistulas) between organs:** protein loss due to severe inflammation and excessive levels of TNF-α - **Overgrowth of bacteria in the gut affecting protein absorption:** changes in microbiota, alterations in lipid digestion and steatorrhea
**Increased metabolism**	- **Elevated levels of pro-inflammatory cytokines:** the levels of inflammatory cytokines, including TNF-α, IFN-γ, IL-1β, IL-6, and IL-1, are elevated in the intestines of IBD patients and are responsible for an increased basal metabolism. - **Increased energy expenditure due to the body’s response to inflammation:** energy is stored mainly as fat in adipose tissue. A negative fat balance increases the lipid mobilization and has been closely correlated with emaciation. - **Administration of corticosteroids affecting metabolism:** increased protein turnover and increased energy expenditure - **Complications from infections affecting metabolism:** increased basal metabolism due to increased pro-inflammatory cytokines

**Table 2:** The main micronutrient deficiencies in IBD, consequences, and recommendations.

Micronutrient	Consequences	Recommendations
Iron	Anemia, fatigue, weakness, brittle nails	Iron deficiency anemia: supplement with oral iron if mild anemia, inactive IBD and good oral tolerance; supplement with intravenous iron if hemoglobin <10 g/dL, active IBD or oral iron intolerance Iron deficiency without anemia: supplement with oral iron
Vitamin D	Disturbed calcium homeostasis and bone health	Supplement in case of detecting deficit
Vitamin B12	Anemia, fatigue, neurological effects	Supplement in case of detecting deficit and in all patients with ileal resection >20 cm
Zinc	Impaired healing, disturbed smell and taste, delayed growth in children	Supplement in case of detecting deficit
Folate	Anemia, fatigue	Supplement in case of detecting deficit
Calcium	Decreased bone density (risk of bone fracture)	Supplement in case of detecting deficit
Magnesium	Disturbed bone health, muscular cramps, fatigue	Supplement in case of detecting deficit

**Table 3:** Description of diets reviewed in the article.

Dietary Pattern	Description of Diet	Evidence of Efficacy
**Crohn’s Disease Exclusion Diet**	Whole foods diet in conjunction with partial enteral nutrition. Eliminates dairy products, processed foods, preservatives, products containing emulsifiers, artificial sweeteners, coffee, alcohol, certain animal fats, and minimizes gluten	RCT in 44 adults with and without PEN showing induction of remission and mucosal healing in some [49] Observational study on 96 patients with and without PEN showing induction of remission and remission maintenance [50]
**Plant-based Diet**	Diet that eliminates or minimizes non-plant-based foods; not necessarily entirely vegetarian or vegan.	Observational study showing reduced risk of relapse in 16 patients with Crohn’s disease in clinical remission [51]
**Low-fat Diet**	Reduce fat to 10% of total calories Reduce red meat to approximately half a serving daily Include 25 g fibers	Crossover RCT in 17 patients with ulcerative colitis in remission, a low-fat and high-fiber diet improved inflammation and intestinal dysbiosis [52]
**Low-meat Diet**	Reduce red and processed meat to one serving per month or less	Prospective RCT in 214 patients with Crohn’s disease in remission showed no difference in relapse between low and high meat intakes [53]
**IgG Exclusion Diet**	Restriction of food based on presence of IgG antibodies specific to 14 food antigens, including egg, wheat, milk, corn, rice, soybean, chicken and others	Observational study in 97 UC patients in remission or mild to moderately active disease on open-label IgG exclusion diet or unchanged diet showed lower stool frequency in exclusion group and no endoscopic differences [54]
**Anti-inflammatory Diet**	Eliminates processed carbohydrates, increases n-3-polyunsaturated fatty acids, and decreases n-6-polyunsaturated fatty acids. Examples of disallowed foods include cured meats, fruit juices, and many dairy items.	RCT on 28 UC patients in remission revealed comparable relapse rates and levels of fecal calprotectin [55]
**Exclusion Diet**	Reduces intake of disaccharides, saturated fats, emulsifiers, red meats, and ultra-processed meats	Case-control study on 139 IBD patients showed higher rates of remission in the exclusion group [56]
**Partial Enteral Nutrition**	Replacement of 35–50% of regular food intake with enteral nutrition	Several RCT on CD patients revealed lower rates of relapse in the intervention group [57,58,59,60,61,62]
